# Therapeutics of acute myeloid leukemia with central nervous system involvement

**DOI:** 10.46989/001c.131722

**Published:** 2025-03-11

**Authors:** Edwin U. Suárez, Tamara Castaño-Bonilla, Rocio Salgado, Laura Solán, Alberto Lázaro-García, Juan M Alonso-Domínguez

**Affiliations:** 1 Hematology Hospital Universitario Fundación Jiménez Díaz

**Keywords:** extramedullary manifestation of AML, FLT3, acute myeloid leukemia, refractory central nervous system leukemia, sorafenib

## Abstract

*FLT3*-mutated acute myeloid leukemia (AML) with central nervous system (CNS) involvement poses therapeutic challenges. We describe two cases and performed a systematic review evaluating the efficacy of therapeutic strategies in CNS involvement for both *FLT3*-mutated and wild-type (WT) AML. A MEDLINE, EMBASE, and Cochrane literature search identified relevant studies. Although CNS involvement in AML is associated with poor prognosis, routine CNS prophylaxis is not standard. Due to the uncertainty regarding the effect of intermediate doses of cytarabine on CNS involvement, we support a diagnostic lumbar puncture (LP) after achieving complete remission in patients with risk factors for CNS infiltration. Consolidation management should be modified depending on the result of the LP. The impact of total body irradiation (TBI) as a conditioning regimen in allogeneic stem cell transplantation on CNS AML outcomes remains ambiguous. Routine craniospinal irradiation is not recommended due to its associated higher morbidity rates, while cranial radiotherapy is preferred, particularly when combined with TBI. Fortunately, currently we can employ a FLT3 inhibitor with CNS penetrance in *FLT3*-mutated (either gilteritinib or sorafenib) or *FLT3*-WT (sorafenib) AML patients.

## Introduction

We describe two *FLT3*-mutated acute myeloid leukemia (AML) cases with central nervous system (CNS) involvement. We performed a systematic review of the literature questioning the effectiveness of the main therapeutic strategies in CNS involvement in AML, including either studies for *FLT3*-ITD mutated or wild type (WT) AML.

## Cases

### Case 1

A 41-year-old man was diagnosed with AML with biallelic *CEBPA* mutation. A massive infiltration of bone marrow (BM) was detected with 80% of blasts. Cytogenetic analysis showed a normal karyotype in 20 metaphases and no other molecular alterations were detected in the next generation sequencing analysis. He was treated with a 7+3 induction regimen (with idarubicin and cytarabine [Ara-C]), achieving complete remission (CR), with negative minimal residual disease (MRD), as assessed by multiparameter flow cytometry (MFC). He was next treated with Ara-C (3g/m^2^/12h) on days +1, +3, +5, as consolidation, for two cycles. Twelve months later, a relapse was identified with 84% blasts in the peripheral blood. Karyotype remained normal. At that time, a *FLT3*-ITD mutation was detected alongside the biallelic mutation of *CEPBA*. He received a FLAG-Ida regimen (fludarabine cytarabine idarubicin and filgrastim), achieving a second CR, followed by Ara-C (2 g/m^2^/12h) on days +1, +3, +5. On day+39 of consolidation, recovered from aplasia, he presented with pain in the pelvis and lower extremities, as well as weakness and paresthesia in the first toe of both feet and involvement of the right cranial nerve (VII). Complete blood count was normal (leukocytes 3.5 x 10^9^/L, hemoglobin 11.9 g/dL and platelets 237x 10^9^/L, without blasts). Brain and thoracolumbar spine magnetic resonance imaging (MRI) showed signs of leptomeningeal infiltration in the roots of the cauda equina. Consequently, lumbar puncture (LP) was performed, which showed cerebrospinal fluid (CSF) with 12 x 10^9^/L cells with 99% cellularity by MFC, compatible with blast cells. BM aspirate showed 2.7% blasts by cytology and MFC showed 4% cells with an aberrant immunophenotype as found at diagnosis. The *FLT3*-ITD mutation was detected in the CNS by polymerase chain reaction (PCR) and fragment length analysis with the same size detected in the BM. He received 4 cycles of intrathecal (IT) chemotherapy with methotrexate (12 mg), Ara-C (30 mg) and dexamethasone (4 mg). Additionally, he received two cycles of systemic methotrexate (2g/m^2^) and Ara-C (2g/m^2^/12h), oral sorafenib (400 mg/12h) and whole-cranial and spinal radiotherapy (RT). At the end of the treatment, with negative MRD by MFC and neurological improvement with disappearance of disease in the CSF, haploidentical allogeneic stem cell transplantation (alloSCT) from his cousin was performed, without major complications. He still receives maintenance treatment with sorafenib and at last follow-up (3.5 years post-alloSCT) is alive and with no signs of relapse.

### Case 2

A 56-year-old man was diagnosed with AML with *NPM1* mutation. Additionally, his AML showed a normal karyotype and *FLT3*-ITD. At diagnosis, he presented with epigastric pain and 10 g/dL of hemoglobin, 55 x 10^9^/L platelets and 120 x 10^9^/L leukocytes, with 75% of blasts and monoblastic cells, with lactate dehydrogenase (LDH) of 965 IU/L. He was treated with a 7+3 protocol and midostaurin, without achieving CR. Reinduction with FLAG-Ida regimen was performed, leading toa first CR with positive MRD detected by allele specific PCR for *NPM1* type A mutation. He received consolidation with Ara-C (1g/m^2^/12h) and midostaurin. On day+35 post-Ara-C, he went to the emergency room due to right retro-ocular headache of several days of evolution, with progressive binocular diplopia and right palpebral ptosis (third cranial nerve involvement). No fundus abnormalities were detected. Brain MRI did not show lesions. MFC detected myeloid blasts in the CSF. BM did not show disease relapse. After 2 cycles of IT chemotherapy with methotrexate (12 mg), Ara-C (30 mg) and dexamethasone (4 mg), craniospinal RT and sorafenib (400 mg/12h, daily), he had a good clinical response, with disappearance of disease in the CSF. Haploidentical alloSCT from a sibling donor was performed, but died at day+10, probably because of respiratory sepsis.

## Discussion

Extramedullary disease (EMD) is a known manifestation of AML, with a reported overall incidence ranging between 0.6 to 24% of the cases.^1^ The most common sites of EMD, in decreasing order of frequency (excluding lymph node, spleen and liver involvement), are soft/connective tissue, skin/breast, gastrointestinal system, bone, head and neck, CNS, and reproductive organs.^1^

CNS involvement in adults with AML is rare, and can be observed both at diagnosis (1.1%) or relapse (2.6 - 4.1%).^2,3^ CNS involvement is more frequent among patients with hyperleukocytosis, elevated LDH, CD56 expression, monocytic AML and those with different genetic features,^1,3^ namely, *FLT3*-ITD mutations, core binding factor leukemias, trisomy 8, *KMT2A* (previously named *MLL*) alterations, complex karyotype or acute promyelocytic leukemia (APL) with *PML::RARA* in relapse.^4,5^

Despite the identification of risk factors for CNS involvement in AML, the standard of care does not include CSF evaluation in the absence of EMD or CNS symptoms, and current treatment does not include CNS prophylaxis with IT chemotherapy.^3^ However, in some clinical trials, LP has been performed in all patients and, interestingly, when conventional cytology (CC) is employed, the frequency of CNS involvement seems to be similar to when it is only performed in the case of CNS symptoms (around 1%).^2^ An additional study also reported on a LP being performed in every patient, and employed CC and MFC to detect leukemic infiltration. Infiltration of CNS by AML was detected in 32% of patients at diagnosis by MFC, with CC detecting only a third of the cases detected by MFC. Interestingly, detection of CNS involvement by MFC at diagnosis showed a significant reduced disease-free survival and overall survival (OS).^6^

Another reason for not performing an LP routinely is the relative low incidence of CNS relapse with the use of high-dose Ara-C (HiDAC) in consolidation therapy, which may have a secondary benefit through its ability to penetrate the blood-brain barrier (BBB).^2,5^ Nevertheless, European LeukemiaNet (ELN) guidelines recommend administering, during consolidation, intermediate doses of Ara-C, which seem to reach a modest CNS concentration, thus having a preventive effect on CNS relapses.^7,8^ Excluding patients with APL, specific guidelines for platelet thresholds in patients with CNS involvement due to leukemic disease remain limited, and most recommendations are extrapolated from other scenarios; for patients with CNS involvement, maintaining platelet counts above 40-50 x 10^9^/L is generally advised, to reduce the risk of CNS hemorrhage in high-risk situations such as LP/IT.^9^

We performed a search on MEDLINE, EMBASE, and the Cochrane Library, using the following terms without time limits: ’’central nervous system”, “central nervous system involvement,” “acute myeloid leukemia’’,”myeloid sarcoma,” “intrathecal chemotherapy,” and “radiotherapy”, to evaluate the efficacy of different therapies for AML with CNS involvement, including studies for *FLT3*-mutated and *FLT3*-WT AML. The bibliographic references of all retrieved studies and reviews were assessed for additional studies. Unpublished works were identified by searching the abstracts of the most important conferences on hematological diseases. The flowchart of analysis of the literature data is reported in Figure 1 (**Supplement 1**). Overall, eleven studies were included in this systematic review (**Table 1**).

**Table 1.** Summary of therapy strategies for CNS involvement in AML (included studies for *FLT3*-ITD mutated or WT AML).

**Intervention, year of publication**	**N of patients; characteristics**	**Outcome(s)**	**Type of study**
Systemic and IT chemotherapy, 2023^5^	52 patients; CNS involvement. 46% of patients *FLT3* mutated.	IT doses preceding CSF clearance, CSF relapse, BM relapse, improvement in neurologic symptoms, and OS.	Retrospective; multicentric.
CNS irradiation as part of the conditioning regimen^10^	5068 AML patients who underwent first alloSCT; 157 patients were CNS “positive” AML. Six patients received CNS irradiation as part of the conditioning regimen.	OS did not significantly differ from that of patients who did not receive CNS irradiation.	Retrospective; multicentric.
Gilteritinib, 2022^11^	1 patient; meningeal and CFS *FLT3*-ITD AML.	Complete regression of meningeal relapse.	Case report.
Sorafenib, 2022^12^	26 patients; refractory CNS acute leukemia (*FLT3*-ITD was negative in 15 patients).	The primary end point was the CR rate within 8 weeks of treatment.	Phase 2 trial.
Sorafenib, 2018^13^	1 patient; *FLT3*-ITD WT R/R AML.	Significant shrink of tumor volume.	Case report.
Sorafenib, 2019^14^	53 patients; 7 patients with pos alloSCT relapsed AML.	Improved remission rates, OS, and PFS for *FLT3*-ITD AML relapsing after alloSCT.	Retrospective; multicentric.
Quizartinib, 2022^15^	1 patient; AML with primary induction failure and CNS involved. Bridge to UCB-SCT.	CR was maintained for 1 year.	Case report.
RT, systemic and IT QT, 2014^16^	34 patients; isolated CNS recurrence.	Effectiveness of the tailored CNS directed treatment modalities (CNS CR, DFS, OS).	Retrospective; unicentric.
Cranial RT, 2011^17^	71 patients; CNS leukemia.	The survival of RT patients was significantly better than IT patients.	Retrospective; unicentric.
RT, 2014^18^	163 patients (66 with AML); pathologic or radiographic evidence of CNS leukemia treated with RT.	Better CNS progression-free survival after RT craniospinal axis/whole brain RT than with skull RT.	Retrospective; unicentric.
IT Ara-C, 1973^19^	9 patients: acute leukemia with CNS involvement.	Improvement of the clinical findings and regression of laboratory evidence of CNS leukemia.	Retrospective; unicentric.

**N:** number; **CNS:** central nervous system; **AML;** acute myeloid leukemia; **R/R:** relapsed / refractory; **alloSCT:** allogeneic stem cell transplantation; **CR** complete remission; **UCB-SCT:** umbilical cord blood stem cell transplantation; **PFS:** progression-free survival; **DFS:** disease-free survival; **OS:** overall survival; **IT:** intrathecal; **CSF:** cerebrospinal fluid; **BM:** bone marrow; **RT:** radiotherapy; **Ara-C**: cytarabine.

IT chemotherapy can clear the CSF of malignant cells, but since this treatment approach, by itself, is not sufficient to prevent relapse, high-dose systemic chemotherapy is also recommended.^3^ RT is usually reserved for patients with symptomatic cranial nerve involvement or for those cases where a tumor mass is impinging on neural structures.^3^ Cranial RT may also be considered for patients who do not respond fully to IT and systemic chemotherapy.^3^ For those who do not respond to conventional therapy or who relapse, there is a lack of standard treatment, and the prognosis is dismal.^4^ Likewise, there have been no prospective studies comparing IT chemotherapy, systemic chemotherapy, and RT in patients with CNS involvement.^3^

In the context of alloSCT, CNS involvement is a prognostic factor but not always determinative: it often correlates with worse outcomes, particularly increased risk of CNS relapse and, maybe, reduced OS.^20,21^ However, it is not uniformly predictive, and outcomes may vary based on other factors (e.g., systemic disease control and use of additional CNS-targeted therapies).^10^ The systemic disease burden at the time of alloSCT is a critical determinant of outcomes, potentially outweighing the impact of CNS involvement in some cases.^22^ There is no recommendation on the use of antiepileptic drugs in this specific context and no contraindication to the use of busulfan in the conditioning regimen. Another important fact is that the incidence and outcomes of patients with CNS relapse are comparable following haploidentical-alloSCT and identical sibling-alloSCT.^20^ The impact of total body irradiation (TBI) on CNS AML outcomes is unclear, with studies showing no significant effect on relapse, relapse-free survival, or OS.^21,22^ Routine craniospinal irradiation is not recommended, due to its increased risk of morbidity. Conversely, cranial RT is preferred, particularly when combined with TBI. The total dose should not exceed 24 Gy.^23^

Several FLT3 inhibitors with variable profiles have been developed in the last few years and have been evaluated in myriad clinical trials as monotherapy or in combination with conventional therapy.^24,25^ Some tyrosine kinase inhibitors such as gilteritinib and sorafenib may penetrate the BBB and have shown promising results for the treatment of CNS leukemic involvement in *FLT3*-ITD AML.^6,11,26^ Furthermore, it appears that sorafenib can also be employed in the case of CNS involvement in *FLT3*-WT AML. This was shown by a recent open label, phase 2 trial, conducted in China, including 26 patients with refractory CNS acute leukemia.^12^ *FLT3*-ITD was negative in 15 patients at both the initial diagnosis and at the time refractory CNS AML was established. They were treated with sorafenib 400 mg orally twice daily. Conventional CNS-directed therapies, such as RT and IT or systemic chemotherapy, were combined with sorafenib therapy according to the investigator’s discretion and the patient’s condition. Additionally, patients with hematological relapse received systemic chemotherapy. When patients achieved CR, sorafenib was administered as maintenance therapy, continuously for 12 months or until the occurrence of relapse or intolerable toxicity. After 8 weeks of treatment, 21 patients achieved CR (80.8%, 95% CI, 62.1% - 91.5%); including 87% (13/15) patients with *FLT3*-WT leukemia. The 2-year event-free survival (EFS) was 75% (95% CI, 54.4%-88.3%) and OS was 76.9% (95% CI, 54.2%-90.4%). Despite the inherent limitations of the study (no comparison group) and the sample size, the use of sorafenib in this setting appears to be safe and effective. Although the activity of FLT3 inhibitors in *FLT3* WT AML patients may seem surprising, the phase 2 QUIWI trial (NCT04107727) designed by the PETHEMA group, has already shown the activity of FLT3 inhibitors in these patients.^27^ It is still unclear whether any specific treatment strategy for *FLT3*-mutated AML can reduce the risk of CNS relapse after alloSCT; while previous studies have demonstrated the intracranial activity of both sorafenib and gilteritinib, the rates of CNS recurrence post-alloSCT have not been reported in either of these trials.^27,28^ We believe that, to have some evidence in this topic, patients diagnosed with AML and presenting with CNS involvement should not be excluded from participation in clinical trials.

Given the controversy over the effect on CNS of intermediate doses of Ara-C, we support the National Comprehensive Cancer Network guideline strategy of performing a diagnostic LP once CR is achieved in those patients with risk features for CNS infiltration (monocytic differentiation, *FLT3*-ITD).^29^ A sensitive technique such as MFC should be employed to select patients with low probability of CNS relapse who would receive intermediate doses in consolidation.^6^ Patients with detectable AML in CNS by MFC could benefit from receiving HiDAC to decrease the risk of CNS relapse.

Fortunately, apart from the already known effective therapies for AML infiltration (i.e., IT chemotherapy, systemic chemotherapy and RT), we currently have the option of employing a FLT3 inhibitor in *FLT3*-mutated (either gilteritinib or sorafenib) or *FLT3*-WT (sorafenib) AML patients.

### Authors’ Contribution

Conceptualization: Edwin Uriel Suárez, Juan M. Alonso-Domínguez; Methodology: Edwin U. Suárez, Juan M. Alonso-Domínguez; Formal analysis and investigation: Edwin U. Suárez, Juan M. Alonso-Domínguez; Writing - original draft preparation: Edwin U. Suárez, Juan M. Alonso-Domínguez; Writing - review and editing: Edwin U. Suárez, Juan M. Alonso-Domínguez, Laura Solán, Tamara Castaño-Bonilla, Rocío Salgado, Alberto Lázaro-García; Resources: Edwin U. Suárez; Supervision: Juan M. Alonso-Domínguez.

### Competing of Interest – COPE

No competing interests were disclosed.

### Ethical Conduct Approval – Helsinki – IACUC

This study was conducted in accordance with the Code of Ethics of the World Medical Association (Declaration of Helsinki). All procedures were performed in an ethical manner, respecting the privacy rights of human subjects. Patients gave their informed consent to use clinical data for this purpose. Patients gave their informed consent to use clinical data for this purpose.

### Informed Consent Statement

All authors and institutions have confirmed this manuscript for publication.

### Data Availability Statement

All are available upon reasonable request.

## Supplementary Material

Supplement 1.
