# Supplementary material for: Therapeutics of acute myeloid leukemia with central nervous system involvement
Source: Clin Hematol Int. 2025 Mar 11;7(1):40–6. doi: 10.46989/001c.131722 (PMC11906164; doi:10.46989/001c.131722)
Supplement: Supplement 1. [file chi_2025_7_1_131722_269966.docx]

















*From:*  Page MJ, McKenzie JE, Bossuyt PM, Boutron I, Hoffmann TC, Mulrow CD, et al. The PRISMA 2020 statement: an updated guideline for reporting systematic reviews. BMJ 2021;372: n71.  <https://doi.org/10.1136/bmj.n71>
